# Trends in developing nanoparticle-enhanced electrochemical sensors for neurotransmitter detection: a review

**DOI:** 10.5114/bta/221513

**Published:** 2026-06-27

**Authors:** Sulastri Sulastri, Ahyar Ahmad, Hasnah Natsir, Isra Wahid, Abdul Karim, Wahyudin Rauf, Harningsih Karim

**Affiliations:** 1 Doctoral Program, Department of Chemistry, Faculty of Mathematics and Natural Science, Hasanuddin University, Makassar, Indonesia; 2 Department of Pharmacy, Faculty of Mathematics and Natural Sciences, Pancasakti University, Makassar, Indonesia; 3 Department of Chemistry, Faculty of Mathematics and Natural Science, Hasanuddin University, Makassar, Indonesia; 4 Research and Development Center for Biopolymers and Bioproducts, Institute for Research and Community Service, Hasanuddin University, Tamalanrea, Makassar, Indonesia; 5 Faculty of Medicine, Hasanuddin University, Makassar, Indonesia; 6 Department of Pharmacy, School of Pharmacy, Yamasi, Makassar, Indonesia

**Keywords:** nano-biosensors, nanotechnology, biomolecules, nanoparticles, electrochemistry

## Abstract

Nano-biosensors represent innovative analytical devices that couple nanotechnology with biomolecular recognition elements to detect specific targets with high sensitivity and selectivity. By incorporating nanomaterials such as gold, carbon, or metal oxides, these devices exhibit enhanced conductivity, larger surface areas, and improved electron transfer, thereby enabling rapid and accurate detection even at ultralow concentrations. Electrochemical nano-biosensors are particularly advantageous for medical diagnostics, health monitoring, and environmental applications because they convert bioreceptor–analyte interactions into measurable real-time electrical signals. Due to their ability to monitor key biomarkers, including neurotransmitters, these sensors hold immense promise for early disease diagnosis, therapeutic monitoring, and neurological research. This review highlights current trends in the development of nanomaterial-enhanced electrochemical sensors for neurotransmitter detection, focuses on their performance and clinical translation potential, and outlines future directions to address challenges in selectivity, stability, and large-scale manufacturing.

## Introduction

Neurotransmitters play a crucial role in mediating various physiological and pathological processes within the nervous system, including the regulation of emotions, cognition, and responses to external stimuli (Teleanu et al. 2022). Imbalances in neurotransmitter signaling are fundamentally linked to major neurological disorders, including Parkinson’s disease, Alzheimer’s disease, depression, and schizophrenia. Furthermore, gaseous neurotransmitters such as nitric oxide (NO) play major roles in neuromodulation and exhibit protective effects, including neuro-renal preservation under certain pathological conditions (Habibey et al. 2010). Consequently, the rapid, accurate, and highly sensitive detection of neurotransmitters is vital for medical diagnostics, therapeutic monitoring, and neurological research (Govindaraju et al. 2023; Li et al. 2019; Teleanu et al. 2022).

Traditional analytical methods for tracking neurotransmitters, including mass spectrometry, fluorimetry, chemiluminescence, and chromatography, offer high accuracy but present significant limitations in clinical applications. These modalities are expensive, require specialized personnel, involve lengthy sample processing procedures, and cannot support real-time monitoring or point-of-care diagnostics (Chauhan et al. 2020). This growing demand for rapid, cost-effective diagnostic tools has accelerated the development of alternative sensing platforms capable of providing immediate results without compromising sensitivity or selectivity. Ultimately, early and accurate detection of neurotransmitter imbalances allows clinicians to identify pathological conditions before symptoms manifest (Ardila 2025).

Electrochemical sensors have emerged as highly promising analytical tools for neurotransmitter detection due to their inherent cost-effectiveness, rapid response times, miniaturization potential, and compatibility with multiplexed detection (Madhurantakam et al. 2020; Ribeiro et al. 2016; Sengupta 2025). However, when employed for neurotransmitter detection, conventional electrochemical sensors face significant challenges, particularly regarding selectivity within complex biological matrices, sensitivity at physiological concentrations, and long-term stability in biological environments. Given the structural similarities among biogenic amines, achieving high selectivity is important, especially when analyzing complex biological fluids containing multiple neurotransmitters (Eskandarinezhad et al. 2022).

The integration of nanomaterials into electrochemical sensing platforms has revolutionized neurotransmitter detection (Fritea et al. 2021). Nanomaterials, including metal nanoparticles (MNPs; gold, platinum, silver), carbon-based materials (graphene, carbon nanotubes [CNTs], carbon dots), and metal oxide nanoparticles, are characterized by unique properties that address the fundamental limitations of conventional sensors. These materials exhibit enhanced electrical conductivity, increased surface areas for biomolecular interactions, improved electron-transfer kinetics, and versatile opportunities for surface functionalization with specific recognition elements. However, there are few comparative studies assessing the relative effectiveness of different nanomaterials and how their unique properties can be leveraged to detect specific neurotransmitters (Power et al. 2018; Zhu et al. 2015).

Despite a surge in the literature regarding nano-enhanced electrochemical sensors, several critical gaps remain. Although numerous reviews have discussed the application of nanomaterials in biosensors, significant challenges in designing electrochemical sensors for neurotransmitters remain to be addressed. First, systematic comparative analyses of different nanomaterial platforms are scarce, making it difficult to identify optimal materials for specific neurotransmitter targets. Second, the rationale for nanomaterial selection is often inadequately justified, with insufficient consideration of trade-offs between sensitivity, selectivity, stability, and cost. Third, translating laboratory prototypes into clinically viable devices remains challenging, with limited discussion of regulatory pathways, manufacturing scalability, and real-world performance validation.

The integration of biomolecules, such as enzymes, into electrochemical biosensors significantly enhances selectivity and enables the detection of non-electroactive neurotransmitters (Tong et al. 2022). In the rapidly evolving field of biomedical engineering, aptamer-based electrochemical sensors have gained increasing attention owing to their high specificity and sensitivity in biomolecular detection (Reaño and Escobar 2024; Urmi et al. 2024). Aptamers are short DNA or RNA sequences with high affinity for target molecules, offering superior selectivity over conventional electrochemical methods. Another emerging approach involves molecularly imprinted polymer (MIP)-based electrochemical sensors, which offer precise molecular recognition through synthetic polymer cavities tailored to specific target molecules (Mohsenzadeh et al. 2024).

## Scope and objectives

This review addresses the gaps in the literature by providing a comprehensive analysis of nanomaterial-enhanced electrochemical sensors for neurotransmitter detection. Specifically, the review evaluates current trends in the development of nanoparticle-based electrochemical sensors for neurotransmitter detection, focusing on the associated challenges, advantages, and future directions. Our primary objectives are to: (1) systematically compare the key performance matrices (sensitivity, selectivity, linear range, detection limit) of different nanomaterial platforms across major neurotransmitter classes; (2) critically assess the advantages and limitations of various nanomaterial approaches, including technical challenges related to reproducibility, stability, and interference management; (3) evaluate technology readiness by assessing the clinical translation potential of different sensor technologies, considering regulatory requirements, manufacturing feasibility, and real-world applicability; (4) identify future directions by highlighting specific research gaps and promising emerging technologies that warrant further investigation.

This review also discusses recent advances in the development of electrochemical sensors for neurotransmitter detection, focusing on studies published in the past decade describing direct electrochemical measurements as well as enzyme-mediated detection strategies. Furthermore, it evaluates progress in the *in vitro, in vivo*, and *ex vivo* applications of neurotransmitter biosensors (Reaño and Escobar 2024; Teleanu et al. 2022).

## Neurotransmitter classification and detection challenges

Neurotransmitters are systematically classified by chemical structure and biological function (Table 1). This classification is essential for understanding detection challenges, as different neurotransmitter classes possess distinct analytical requirements.

**Table 1:** Classification of neurotransmitters, biological functions, and chemical structures

Category	Neurotransmitter	Biological functions	Chemical structure
Amino acids	Glutamate	Memory and learning	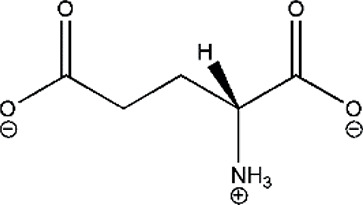
	Tyrosine	Regulation of energy balance, memory, and learning	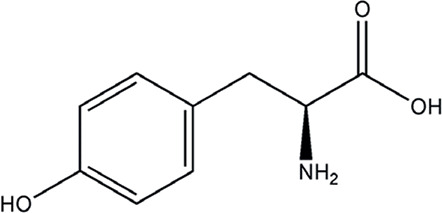
Biogenic amines	Dopamine	Responsible for feelings of pleasure	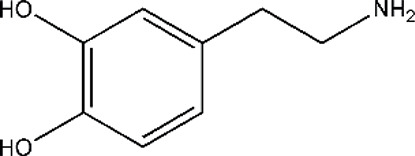
	Epinephrine	Leads to physical arousal and increased awareness	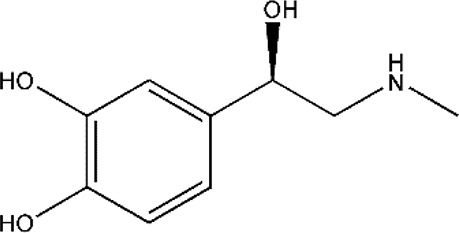
	Norepinephrine	Enhances attention and responsiveness	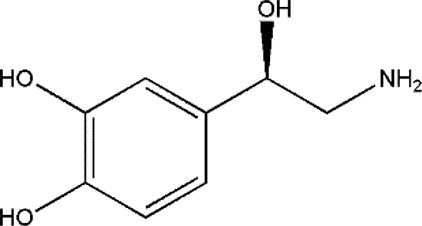
	Serotonin	Regulates mood, sleep, emesis, sexuality, appetite	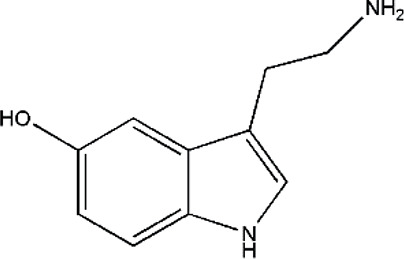
	Tryptamine	Acts on the central nervous system and digestive tract	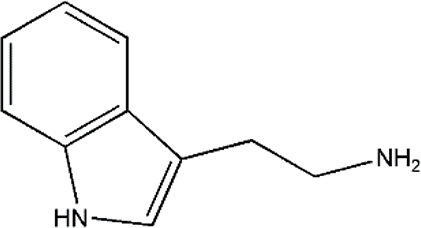
Acetylcholine	Acetylcholine	Cognition, learning, and memory	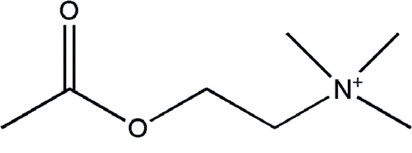
Soluble gases	Nitric oxide	Cognitive function, homeostatic function, neurosecretion, and synaptic plasticity	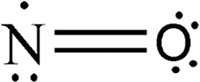
	Hydrogen sulfide	Neuromodulator in the brain	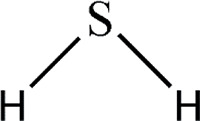

Accurate detection of these biomarkers in various biological fluids is essential for disease diagnosis, progression monitoring, and therapeutic guidance. Although various analytical methods have been developed for neurotransmitter analysis, including mass spectrometry, fluorimetry, chemiluminescence, chromatography, and capillary electrophoresis, their clinical utility remains limited. These methods are often expensive, require highly trained personnel, and involve lengthy processing times before delivering results, making them impractical for real-time and on-site monitoring (Chauhan et al. 2020). To overcome these limitations and enhance patient care, clinical laboratories continuously strive to develop reliable, cost-effective, and rapid diagnostic platforms while optimizing existing protocols for efficiency (Ardila 2025; Janicot et al. 2024). Ultimately, early and accurate detection of neurotransmitter imbalances is vital for identifying underlying pathological conditions before clinical symptoms manifest (Ardila 2025; Ellison et al. 2025).

Electrochemical sensors have been proposed as a promising alternative for neurotransmitter detection due to their cost-effectiveness, rapid response times, and ability to simultaneously detect multiple analytes (Sengupta 2025). These sensors are particularly valuable for real-time and point-of-care applications, allowing for seamless detection even by untrained individuals. However, given the structural similarities among biogenic amines, maintaining selectivity remains a primary hurdle, especially when analyzing complex biological fluids containing multiple neurotransmitters (Eskandarinezhad et al. 2022).

The integration of bio-elements, such as enzymes, into electrochemical biosensors significantly enhances selectivity by mitigating matrix interference, which enables the detection of non-electroactive neurotransmitters. This review discusses recent advances in the development of electrochemical sensors for neurotransmitter detection, focusing on studies published over the past decade that describe direct electrochemical measurements and enzyme-mediated detection strategies. Furthermore, it evaluates progress in the *in vitro*, *in vivo*, and *ex vivo* applications of neurotransmitter biosensors (Reaño and Escobar 2024; Teleanu et al. 2022).

In the rapidly evolving field of biomedical technology, aptamer-based electrochemical sensors have attracted increasing attention for their high specificity and sensitivity in biomolecular detection (Reaño and Escobar 2024; Urmi et al. 2024). Aptamers are short DNA or RNA sequences with a strong affinity toward target molecules, providing superior selectivity over conventional electrochemical interfaces. Aptasensors utilize these aptamers as recognition elements for the rapid and easy detection of proteins. Based on their signal transduction mechanisms, electrochemical aptasensors can be categorized as labeled or label-free. Labeled variants typically incorporate redox-active molecules, nanomaterials, or DNAzymes to achieve robust signal detection.

Another trending approach involves MIP-based electrochemical sensors, which offer precise molecular recognition through synthetic polymer cavities tailored to specific target molecules. In these sensors, MIP-coated electrodes selectively bind to target neurotransmitters, inducing detectable electrochemical changes that enable accurate quantification (Mohsenzadeh et al. 2024).

The integration of nanotechnology has revolutionized neurotransmitter detection by significantly improving the sensitivity, selectivity, and miniaturization of electrochemical sensors. Nanomaterials, such as MNPs, graphene, and CNTs, possess excellent electrical conductivity and large surface areas, facilitating enhanced electron transfer and signal amplification (Barhoum et al. 2024). Carbon-based nanomaterials, including CNTs, graphene, and nanofibers, have demonstrated remarkable mechanical strength and electrochemical properties, both of which are ideal for neurotransmitter biosensors. Further modifications of these materials with functional elements optimize electron transfer processes, facilitating rapid and highly sensitive detection (Fritea et al. 2021; Liu and Zhang 2023; Yang et al. 2016).

Although advancements in nanotechnology, material chemistry, and microfabrication have propelled the development of highly sensitive, selective, and userfriendly electrochemical sensors , several key challenges remain, including sensor stability, interference from biological components, and the need for seamless clinical integration. Future research must focus on improving the reproducibility, portability, and real-time applicability of electrochemical sensors to meet the growing demand for efficient diagnostic tools. To that end, this review explores the latest trends in electrochemical sensor development, focusing on nanomaterial-based strategies and their broader impact on neurotransmitter detection in biomedical applications.

## Literature collection methodology

A systematic literature review was conducted to obtain a comprehensive and structured collection of relevant scientific information. The literature search was performed using a wide range of reputable and well-established online academic databases to capture high-quality, peer-reviewed publications from multidisciplinary fields. Biomedical, biochemical, and life sciences literature was sourced from PubMed and ScienceDirect, while Multidisciplinary Digital Publishing Institute, Springer Link, Wiley, and the American Chemical Society provided access to chemistry, materials science, and applied sciences journals. Additionally, the Royal Society of Chemistry database was included in the search to ensure robust coverage of high-impact chemical research and fundamental mechanistic studies.

To broaden the scope of the review and minimize retrieval bias, databases with extensive citation indexing capabilities, such as Web of Science and Scopus, were systematically searched. This enabled the identification of highly cited articles, recent advances, and influential publications within the research domain. Furthermore, Google Scholar and ResearchGate served as complementary sources to retrieve gray literature, early-access articles, and emerging studies absent from traditional databases. The IEEE Xplore Digital Library database was also searched to obtain technical and engineeringrelated publications relevant to analytical methods, instrumentation, and sensor development.

The literature search strategy was designed to be systematic and reproducible by applying specific keywords, Boolean operators, and inclusion–exclusion criteria tailored to the study objectives. Only articles published in peer-reviewed journals, written in English, and relevant to the research scope were considered for further analysis. Duplicate records were carefully screened and removed, and the selected studies were critically evaluated based on their methodological quality, thematic relevance, and scientific contribution. This structured approach ensured that the literature review was both comprehensive and reliable, providing a solid theoretical foundation and contextual background for the subsequent sections of the study.

### Inclusion and exclusion criteria

The selected articles included original research publications and reviews focused on the development of electrochemical sensors for neurotransmitter detection. The search was restricted to English-language publications from 2015 to 2025 that provided accessible datasets or comprehensive reviews. Publications lacking sufficient experimental information or consisting solely of brief opinions were excluded from the analysis.

### Search strategy

The literature search was conducted using a combination of the following keywords: “electrochemical sensor,” “neurotransmitter detection,” “biosensor,” “aptasensor,” “molecularly imprinted polymers (MIPs),” and “point-ofcare diagnostics.” Subsequently, Boolean operators were applied to refine or expand the search strings, such as (“electrochemical sensor” AND “neurotransmitter”) or (“biosensor” OR “neurochemical detection”).

### Selection process and data analysis

A comprehensive analysis of the reviewed literature revealed several critical challenges in the design, development, and practical application of electrochemical sensors for neurotransmitter detection. A primary challenge is structural instability, as sensor materials are susceptible to degradation over time due to prolonged electrochemical cycling, exposure to complex biological environments, and surface fouling. This degradation causes signal drift, reduced sensitivity, and compromised detection accuracy, thereby limiting the long-term reliability of sensors, particularly for continuous or repeated measurements in clinical and real-world settings.

Another major challenge is selectivity. Neurotransmitters typically coexist in biological fluids with a wide range of electroactive species, such as ascorbic acid, uric acid, glucose, and proteins, which can interfere with the electrochemical signal. The overlapping oxidation or reduction potentials of these interfering substances make it difficult to distinguish the target neurotransmitter with high specificity. Consequently, insufficient selectivity leads to false-positive or false-negative results, significantly reducing the reliability and diagnostic utility of electrochemical sensors in complex biological matrices such as blood, cerebrospinal fluid, or brain tissue.

Scalability of production also remains a significant obstacle to the widespread adoption of electrochemical sensors. Although many sensor designs demonstrate excellent performance at the laboratory scale, their large-scale manufacturing poses considerable challenges. Achieving consistent quality, reproducibility, and performance across mass-produced devices requires precise control over material synthesis, electrode modification, and fabrication processes. Moreover, the use of advanced nanomaterials or complex fabrication techniques can increase production costs, hindering the development of affordable sensors for routine clinical use or commercial purposes.

In addition to manufacturing challenges, the clinical application of electrochemical sensors is further limited by the demands of miniaturization and system integration. Clinical and point-of-care applications require sensors to be compact, portable, and easily integrated with diagnostic devices or wearable platforms. However, miniaturizing electrochemical sensors without compromising sensitivity and accuracy remains technically demanding. Furthermore, integrating these sensors with electronic readout systems, data processing units, and user-friendly interfaces that comply with clinical standards and regulatory requirements adds another layer of complexity to their application.

Despite these challenges, recent innovations demonstrate significant potential to overcome several existing limitations of electrochemical sensors. The incorporation of nanomaterials, such as CNTs, graphene, MNPs, and metal–organic frameworks, has been shown to substantially enhance sensor sensitivity, stability, and surface area, thereby improving signal strength and detection limits (Fritea et al. 2021; Madhurantakam et al. 2020). Additionally, the use of aptamers as recognition elements improves selectivity toward specific neurotransmitters due to their strong binding affinity and molecular specificity (Reaño and Escobar 2024; Urmi et al. 2024), while MIPs provide synthetic recognition sites that mimic biological receptors and offer improved chemical stability and reusability (Mohsenzadeh et al. 2024). Overall, the reviewed literature indicates that ongoing technological advancements are steadily addressing the challenges related to the stability, selectivity, scalability, and clinical applicability of electrochemical. sensors for neurotransmitter detection. Specifically, the integration of novel materials and innovative sensing strategies continuously drives electrochemical sensors to become reliable, cost-effective, and clinically viable solutions for neurotransmitter monitoring in both research and medical settings.

## Electrochemical and mechanism of sensors

Electrochemical sensing technique is widely used for detecting biological and chemical analytes due to its high sensitivity, rapid response, low cost, and compatibility with, miniaturized devices. Recent advancements in nanomaterials, including MNPs, carbon-based nanostructures, and conducting polymers, have further enhanced the overall performance of electrochemical sensors by significantly improving electrode conductivity, surface area, and electron-transfer kinetics. Modern electrochemical sensors work through fundamental mechanisms such as voltammetric, amperometric, potentiometric, and impedimetric responses, which enable the quantification of analytes in complex biological matrices. In recent years, the integration of nanostructured materials with emerging platforms, such as flexible electrodes, wearable biosensors, and microfluidic electrochemical systems, has expanded their utility in biomedical monitoring, environmental analysis, and food safety screening. Therefore, understanding the latest electrochemical principles and sensor mechanisms is crucial for developing highly selective and reproducible detection systems (Reaño and Escobar 2024; Teleanu et al. 2022).

### Comparative advantages of electrochemical sensing platforms

Electrochemical sensors outperform optical, piezoelectric, acoustic, gravimetric, magnetic, and calorimetric alternatives, offering high sensitivity, cost-effectiveness, portability, and microfabrication compatibility in clinical applications (Sanghavi et al. 2015). These sensors are particularly valuable for the detection of neurological biomarkers, where target concentrations often exist at nanomolar to micromolar levels in biological fluids.

#### Electrode modification strategies: a comparative analysis

The selection of an appropriate electrode modification strategy is important for improving the overall performance of electrochemical sensors, particularly regarding sensitivity, selectivity, stability, and response times. Different modification approaches distinctly influence the physicochemical properties of the electrode surface, including surface area, conductivity, catalytic activity, and interactions with target analytes. In this context, a comparative evaluation of dominant electrode modification strategies is essential to understand their advantages and limitations. As summarized in Table 2, conductive polymers (CPs), MNPs, and hybrid materials, which represent the most common modification approaches for neurotransmitter sensing, distinctly enhance electrochemical performance and address specific analytical challenges.

**Table 2:** Comparative performance of electrode modification strategies

Material type	Detection limit range	Linear range	Key advantages	Major limitations	Cost	References
Conductive polymers	0.1–10 μM	1–1000 μM	Interference rejection, tunable thickness	Limited stability	Low	(Shahid et al. 2025)
Gold nanoparticles	0.01–1 μM	0.1–500 μM	Easy functionalization, high conductivity	High cost, aggregation	High	(Chauhan et al. 2025)
Metal nanoparticles	0.1–5 μM	1–200 μM	Large surface area, enzyme immobilization	Magnetic interference	Medium	(Chen et al. 2025)
Hybrid materials	0.001–0.1 μM	0.01–100 μM	Synergistic effects, enhanced sensitivity	Complex synthesis	High	(Govindaraju et al. 2023)

#### CPs vs. MNPs vs. hybrid materials

CPs, MNPs, and hybrid materials differ fundamentally in their modes of action and mechanisms of performance enhancement. CPs primarily improve sensor performance by offering tunable surface chemistry and controlled film thickness, and allowing selective interactions with target molecules (Shahid et al. 2025). In contrast, MNPs enhance electrochemical responses mainly by increasing surface area, improving electron-transfer kinetics, and providing catalytic activity (Chauhan et al. 2025; Chen et al. 2025). Hybrid materials, in which CPs are combined with MNPs or other nanostructures, synergistically integrate the advantages of both materials, thereby overcoming the intrinsic limitations of single-component systems (Govindaraju et al. 2023). The comparative analysis presented in Table 2 highlights how these materials differ in fabrication complexity, cost, reproducibility, and suitability for specific sensing applications.

### CPs: controlled modification strategy

CPs represent a highly controlled and versatile electrode-modifying material, largely due to their ability to be deposited directly onto electrode surfaces via in situ electrodeposition techniques. This approach allows researchers to precisely regulate the thickness, morphology, and uniformity of polymer films by adjusting key electrochemical parameters, such as potential, current density, and deposition time. Such control is particularly advantageous for microelectrode and miniaturized sensor applications, in which consistent surface modification is critical for reproducible performance. Moreover, CPs can be conformally coated onto electrodes with unconventional geometries, expanding their applicability to advanced sensor designs (Shahid et al. 2025).

The electrochemical performance of CP-modified electrodes is strongly influenced by the balance between electron transfer at the electrode–polymer interface and charge transport within the polymer matrix itself. Excessively thick polymer films may hinder electron transfer, whereas overly thin layers may compromise selectivity and stability. Therefore, optimizing this balance is essential for achieving high sensitivity and fast response times. Studies show that carefully engineered CP films significantly enhance signal amplification while maintaining efficient charge transport pathways, thereby improving overall sensor performance (Shoyiga and Fayemi 2025).

A key advantage of CP films is their inherent ability to reduce interference from electroactive species commonly present in biological fluids. Through electrostatic repulsion mechanisms, CPs can effectively minimize the influence of negatively charged interferents, such as ascorbic acid and uric acid, contributing to improved selectivity toward positively charged neurotransmitters. Additionally, diverse monomeric building blocks, including aniline, hydroxybenzoic acid, pyrrole, and methylene blue, allow fine-tuning of polymer conductivity, hydrophilicity, and molecular recognition. This structural flexibility makes CPs highly adaptable to different sensing requirements and target analytes.

Furthermore, electrodeposition techniques enable precise fabrication of uniform and reproducible polymer films, which is particularly beneficial for microelectrode and array-based sensor platforms. Recent reviews emphasize the robustness, tunability, and biocompatibility of CP-based electrochemical sensors for neurotransmitter detection (Bashar and Wing 2020; Elugoke et al. 2020; Sanghavi et al. 2015). These studies collectively highlight CPs as a mature and reliable modification strategy for advanced electrochemical sensing applications (Carbone et al. 2025; Chen et al. 2025).

#### MNPs: enhanced electrochemical properties

MNPs have emerged as one of the most widely adopted nanomaterial-based electrode modification strategies due to their exceptional electrochemical properties and relative ease of fabrication. Incorporating electrode surfaces with MNPs, such as gold, silver, platinum, or copper, significantly increases their effective surface area, and subsequently the number of active sites available for electrochemical reactions. This enhancement leads to improved sensitivity, lower detection limits, and faster electron-transfer kinetics, which make MNP-modified electrodes particularly attractive for neurotransmitter sensing applications.

In addition to electrochemical advantages, MNPs offer considerable versatility in fabrication methods. Techniques such as drop-casting, electrodeposition, and electrochemical reduction enable straightforward and scalable modification of electrode surfaces without requiring complex instrumentation. These methods allow precise control over nanoparticle size, distribution, and surface coverage, which significantly influence sensor performance (Chauhan et al. 2025; Ernis 2025; Gopika and Saraswathyamma 2025). However, despite their high sensitivity, MNP-based sensors frequently exhibit low long-term stability and selectivity, particularly in complex biological matrices.

Overall, the comparative analysis of CPs, MNPs, and hybrid materials underscores the importance of selecting an electrode modification strategy that aligns with the specific performance requirements of neurotransmitter detection. While CPs allow controlled surface modification and offer selectivity, MNPs show superior electrochemical activity and signal amplification. Hybrid materials, by integrating these complementary properties, appear promising for the development of next-generation electrochemical sensors.

#### Performance considerations

The performance of nanoparticle-based electrochemical sensors is strongly influenced by the physicochemical properties of the nanomaterials employed. Variations in particle size, surface chemistry, functionalization capacity, and magnetic behavior can significantly affect the sensitivity, selectivity, and overall efficiency of the sensing system. According to recent studies, the synthesis method plays an important role in determining nanoparticle size, which directly influences electron-transfer kinetics and detection performance. Additionally, specific nanomaterials like gold nanoparticles (AuNPs) and metal nanoparticles enhance sensor functionality by improving biomolecular conjugation and enzyme immobilization. These key performancerelated characteristics are summarized in Table 3.

**Table 3:** Characteristics of nanoparticles based on type and sensitivity

Type/factor	Key characteristics	Implications for sensitivity/application
Size-dependent variations	Sensor sensitivity varies according to nanoparticle size, influenced by the synthesis method	Smaller size → larger surface area → enhanced electron transfer and higher sensor sensitivity
Gold nanoparticles	Exhibit excellent chemical and biological modification capabilities	Facilitate biomolecule conjugation; improve selectivity and stability of sensors
Metal nanoparticles	High magnetic susceptibility enabling easy enzyme immobilization; also show peroxidase-mimetic activity	Enable rapid separation and increase efficiency of enzyme-based and catalytic sensors

### Hybrid materials: synergistic performance enhancement

Advanced hybrid materials, in which nanostructured CPs are combined with organic or inorganic components, exhibit precisely controlled compositions and characteristics. These materials demonstrate superior performance through enhanced selectivity, increased sensitivity, expanded active sites, improved uniformity, and stronger electrode surface adherence (Chauhan et al. 2025; Chen et al. 2025; Govindaraju et al. 2023; Moulahoum and Ghorbanizamani 2024).

## Fundamental mechanisms in electrochemical neurotransmitter detection

### Redox reactions: the core detection mechanism

Electrochemical sensors operate by converting biochemical reactions into measurable electrical signals through three primary mechanisms.

### Oxidation reactions

Neurotransmitters lose electrons at the electrode surface, generating proportional current responses.


$$\text{Dopamine}\to {\text{Dopamine}}^{+}+{\text{e}}^{-}$$ (1)

### Reduction reactions

Target molecules gain electrons, a reaction that is highly useful for detecting compounds such as hydrogen peroxide.


$${\text{H}}_{2}{\text{O}}^{2}+2{\text{e}}^{-}\to 2{\text{OH}}^{-}$$ (2)

The efficiency of redox reactions depends mainly on the selection and surface modification of the electrode material, which can be optimized using CPs or MPNs to enchance electron transfer and overall sensor performance (Sahoo et al. 2025).

### Electron transfer efficiency: material-dependent performance

Electron transfer rate and efficiency are pivotal factors that determine sensor responsiveness and sensitivity. The choice of electrode material significantly influences these parameters due to variations in electrical conductivity, corrosion resistance, and target molecule interaction properties (Table 4).

**Table 4:** Electrode material performance comparison

Material	Conductivity	Surface area	Electron transfer rate	Functionalization ease	Stability
Carbon fiber	Moderate	Low	Moderate	Difficult	High
Graphene	Excellent	Very high	Very fast	Moderate	High
Gold	Excellent	Moderate	Fast	Very easy	Moderate
Carbon nanotubes	High	Very high	Fast	Moderate	High

Carbon-based materials, such as carbon fiber and graphite, provide good conductivity, chemical stability, and neurotransmitter adsorption capabilities. CNTs and graphene significantly enhance performance by increasing the surface area and improving electrontransfer kinetics (Gupta et al. 2020; Hwang et al. 2020). Gold electrodes offer excellent conductivity and easy functionalization, which enables rapid electron transfer and enhances selectivity through surface modification (Carbone et al. 2025). Nanomaterials, particularly graphene and metal nanocomposites, accelerate electron transfer by offering outstanding electrical conductivity, large surface areas, and excellent mechanical properties that are ideal for electrochemical applications (Chavan et al. 2025).

### Diffusion and adsorption: transport-limited considerations

Electrochemical signal generation involves a complex interplay between neurotransmitter diffusion to the electrode surface and subsequent adsorption onto the sensor materials (Gopika and Saraswathyamma 2025).

## Critical factors affecting performance

Understanding the key factors that influence sensor performance is essential for improving the detection accuracy, response time, and stability of electrochemical neurotransmitter analysis. Factors – particularly diffusion rate, adsorption efficiency, and adsorption mechanism – directly affect how neurotransmitters migrate toward the electrode surface and how strongly they interact with it. In complex biological environments, rapid diffusion must be balanced with sufficient adsorption to ensure reliable, real-time monitoring. Recent studies emphasize optimizing these interacting parameters to achieve high sensitivity and selectivity in neurotransmitter sensing systems (Alyamni et al. 2024; Ellison et al. 2025). The key factors affecting sensor performance are summarized in Table 5.

**Table 5:** Critical factors affecting sensor performance

Performance factor	Key characteristics	Impact on sensor performance
Diffusion rate	Influenced by neurotransmitter concentration, medium viscosity, and electrode geometry	Determines how quickly analytes reach the electrode surface; affects response time and sensitivity
Adsorption efficiency	Controlled by Van der Waals forces, hydrogen bonding, and electrostatic interactions between the analyte and the electrode	High adsorption enhances signal strength and detection reliability; low adsorption reduces sensitivity
Adsorption type	Physisorption: weak, reversible; chemisorption: strong, stable	Physisorption supports fast kinetics; chemisorption provides stable long-term detection; a balance is needed depending on the application

## Advanced electrochemical techniques: comparative analysis

**Figure 1:**
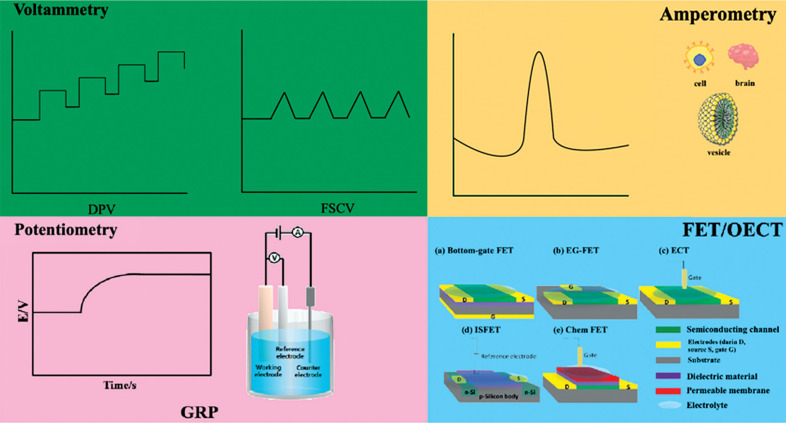
Different electrochemical sensing techniques, including voltammetry, amperometry, potentiometry, field-effect transistors (FET), and organic electrochemical transistors (OECT). Voltammetry and amperometry are shown with examples of their applications in detecting signals from cells, the brain, and vesicles. The image illustrates how these technologies operate across various systems, underscoring the diverse methodologies for in vivo neurochemical sensing. It visually contrasts different signal detection techniques and response times, showcasing their applications in monitoring neurotransmitter activity (Yang et al. 2025). ChemFET – chemical field-effect transistor, DPV – differential pulse voltammetry, ECT – electrochemical transistor, EG-FET – electrolyte-gated field-effect transistor, FSCV – fast-scan cyclic voltammetry, GRP – graphene-related material, ISFET – ion-sensitive field-effect transistor

Yang et al. (2025) reported that in vivo electrochemical sensing technologies are essential tools for real-time neurochemical monitoring, ranging from single cells to entire brain systems. Figure 1 illustrates the fundamental differences between major electrochemical techniques and their application. The Figure 1 shows five distinct electrochemical sensing approaches with complementary advantages:

- voltammetry: a universal technique that allows electrode miniaturization for complex matrix analysis;
- amperometry: provides high temporal resolution, optimal for fast neurochemical kinetics;
- potentiometry: offers quantitative analysis with reduced invasiveness and high biocompatibility;
- field-effect transistors: amplify signals with superior sensitivity;
- organic electrochemical transistors: utilize enhanced amplification strategies that exceed traditional technologies.

The selection of appropriate techniques depends on application requirements: temporal resolution vs. sensitivity vs. invasiveness trade-offs (Yang et al. 2025).

## Performance optimization guidelines

Comparative analysis revealed that optimal sensor design requires:

- material selection: electrode material properties should be matched to target neurotransmitter characteristics;
- modification strategy: polymers, nanoparticles, or hybrid approaches should be chosen based on performance requirements and cost constraints;
- technique selection: the electrochemical method should be selected based on temporal resolution, sensitivity, and invasiveness requirements;
- surface optimization: diffusion and adsorption processes should be balanced for the target application.

Integration of these considerations enables rational sensor design with predictable performance characteristics for the detection of specific neurotransmitters.

## Non-enzymatic electrochemical sensors.

### Overview and comparative advantages

Nonenzymatic (direct or enzyme-free) electrochemical sensors are now preferred for detecting electroactive neurotransmitters, particularly monoamines like dopamine, norepinephrine, and serotonin (Keles et al. 2024; Uçar et al. 2024). Compared to enzymatic counterparts, these sensors offer enhanced stability, simplified fabrication, lower cost, and zero enzyme-related interference. However, selectivity and interference management in complex biological matrices remain challenging.

## Performance analysis and material comparison

### Systematic performance evaluation

Table 6 provides a comprehensive performance comparison of nonenzymatic sensors across various nanomaterial platforms used for detecting major neurotransmitters and integrates previously scattered findings to facilitate rational sensor selection. The comparative analysis highlights several important performance trends, including a clear sensitivity ranking where AuNPs/CNT systems outperform hybrid nanomaterials, molybdenum disulfide/reduced graphene oxide (MoS_2_/rGO) composites, platinum–CNT (Pt-CNT) structures, and graphene-based sensors (Gupta et al. 2020; Nimgampalle et al. 2023). Nonetheless, all platforms exhibit selectivity limitations due to interference from structurally similar molecules in biological matrices. Additionally, a notable performance trade-off is observed, with highly sensitive materials often demonstrating reduced long-term stability, underscoring the importance of carefully balancing these factors, as summarized in Supplementary Table S1.

**Table 6:** Comparative performance of non-enzymatic sensors for neurotransmitter detection

Nanomaterial	Target NT	LOD (μM)	Linear range (μM)	Sensitivity (μA/μM · cm^2^)	Major interferences	Stability (% after 30 days)	References
Graphene/GCE	Dopamine	0.05	1–100	12.5	AA, UA, DOPAC	85%	Cui et al. 2024
AuNPs/CNT	Dopamine	0.01	0.1–50	25.8	AA, UA	90%	Manoharan Nair Sudha Kumari 2024
MoS_2_/rGO	Serotonin	0.08	0.5–80	18.2	Tryptophan, tyrosine	78%	Melo et al. 2025
Pt-CNT	Norepinephrine	0.03	0.2–60	15.6	Dopamine, epinephrine	82%	Bakri et al. 2024
Hybrid polymer/Au	Multiple NTs	0.02–0.15	0.1–200	8.5–22.3	Variable	75–88%	Moulahoum and Ghorbanizamani 2024

AA – ascorbic acid, Au – gold, AuNPs – gold nanoparticles, CNT – carbon nanotubes, DOPAC – 3,4-dihydroxyphenylacetic acid, GCE – glassy carbon electrode, LOD – limit of detection, MoS_2_ – molybdenum disulfide, NT – neurotransmitter, Pt – platinum, rGO – reduced graphene oxide, UA – uric acid.

### Critical assessment of detection strategies

Direct neurotransmitter detection using nanocomposite-modified electrodes represents an effective, highly sensitive strategy. However, the presence of interfering molecules in biological fluids limits the application of these electrodes in real-world analysis (Cui et al. 2024), necessitating careful consideration of strategies to enhance selectivity. The scattered literature results have previously hindered the systematic comparison of detection approaches. This review consolidates comparative data focusing on key parameters, including electrode modifiers (CPs, nanoparticles), linear detection ranges, limits of detection, andsensitivity metrics. These parameters directly determine sensor performance in the detection of electroactive neurotransmitters, which is essential for understanding brain functions and various physiological processes (Sumitha and Xavier 2023).

### Technical challenges and solutions

#### Selectivity enhancement strategies

In neurotransmitter detection, selectivity is often compromised by the presence of structurally similar molecules, overlapping redox potentials, and complex biological matrices that can generate interfering signals. Strategies designed to improve a sensor’s ability to distinguish target analytes from interferents include modifying electrode surfaces with selective nanomaterials, incorporating molecular recognition elements such as aptamers or MIPs, tuning surface charge and functional groups, and applying advanced electrochemical techniques to enhance signal discrimination (Reaño and Escobar 2024; Urmi et al. 2024). Ultimately, selectivity can be improved through material engineering, sensing design optimization, and analytical enhancement methods.

#### Interference management in biological matrices

Interference management in biological matrices is an important parameter in nonenzymatic sensing, largely due to the coexistence of multiple electroactive species within biological samples. Compounds such as uric acid, ascorbic acid, glucose, dopamine, and other metabolites can generate overlapping or competing electrochemical signals that can obscure or distort the response of the target analyte, thereby compromising detection accuracy. Therefore, the applied sensor must be able to identify and minimize interference for achieving reliable and high-performance measurements. Supplementary Table S2 quantifies the interference effects produced by common biological compounds, outlining how each interferent influences the sensor response and the extent to which selectivity can be maintained under realistic experimental conditions.

### Clinical translation assessment

#### Technology readiness evaluation

This evaluation typically examines multiple factors, including analytical robustness, reproducibility, manufacturability, biocompatibility, user safety, and regulatory feasibility. Assessing technology readiness is essential for determining whether a sensing platform can progress from laboratory research to preclinical validation, clinical trials, and subsequent clinical deployment. Supplementary Table S3 provides a structured overview of the technology readiness levels (TRLs) for each sensor platform, highlighting their developmental stages, existing limitations, and the milestones required for successful clinical application.

#### Real-world performance limitations

Supplementary Table S4 provides a systematic analysis of the maturity level of the developed compound or therapeutic candidate before progressing toward clinical application. This evaluation uses the TRL framework to determine research advancement from the basic concept to potential preclinical or clinical implementation.

### Advanced detection approaches

#### Multi-analyte detection systems

Recent scientific developments focus on simultaneously detecting multiple neurotransmitters using advanced array-based sensing approaches (Qi et al. 2025). These technologies outperform traditional single-analyte detection methods, as they allow researchers to monitor several neurochemical signals within the same biological sample. One of the major advantages of array-based systems is their ability to operate with extremely small sample volumes – often < 50 μl – which is particularly valuable in studies involving limited or precious biological materials such as cerebrospinal fluid, microdialysates, or cell culture supernatants. In addition, these platforms enable built-in cross-validation among multiple sensing elements, increasing analytical accuracy and reducing the likelihood of false-positive or false-negative results. The integration of pattern-recognition algorithms further enhances selectivity, enabling systems to distinguish between neurotransmitters with structural similarities or overlapping electrochemical signatures. It also allows real-time monitoring of neurotransmitter ratios, which is crucial for understanding dynamic neurochemical processes, synaptic signaling balance, and the pathophysiology of neurological disorders.

Despite these advantages, array-based systems present challenges that hinder their widespread adoption. The increased complexity of multichannel sensor arrays necessitates more sophisticated device architecture, meticulous calibration, and tightly controlled experimental conditions. These systems are also costly due to the need for precision microfabrication, high-performance detection components, and advanced dataprocessing hardware. Furthermore, the acquisition and interpretation of multivariate signals demand advanced computational resources, including machine learning or chemometric techniques, to extract meaningful patterns from large datasets. Consequently, the successful implementation of array-based systems requires interdisciplinary expertise spanning neuroscience, analytical chemistry, electrical engineering, and data science. Overall, while these systems offer significant promise for improving the resolution and reliability of neurochemical analysis, they must overcome technical and financial barriers before widespread integration into routine research or clinical diagnostics.

#### Integration with digital health platforms

The future of nonenzymatic sensors relies on their integration within advanced digital health ecosystems, necessitating strong data management and regulatory compliance. Effective data management encompasses real-time data transmission, cloud-based pattern recognition, interoperability with electronic health records, and artificial intelligence-assisted interpretation algorithms to enhance diagnostic reliability (Bakri et al. 2024). At the same time, regulatory considerations are essential, including navigating U.S. Food and Drug Administration approval pathways for digital therapeutics, ensuring compliance with data privacy and security frameworks, meeting clinical validation benchmarks, and implementing rigorous quality management systems. These combined requirements form the foundation for translating nonenzymatic sensors into clinically deployable digital health tools, as summarized in Supplementary Table S5.

### Performance optimization guidelines

#### Rational sensor design framework

The development of an effective nonenzymatic sensor requires a rational design framework integrating material properties, performance requirements, and practical constraints. Material selection must align with the intended analytical performance, ensuring sensor sensitivity matches the physiological concentration range of the target analyte. It is also important to ensure high selectivity despite the presence of numerous interfering compounds within biological matrices, which can distort signal accuracy. Stability considerations are also central, as sensors need to retain functionality over the operational duration and under appropriate storage conditions. In addition, economic feasibility must be evaluated to balance high performance with cost-effectiveness, especially for large-scale or clinical deployment.

Design optimization inevitably involves trade-offs. For instance, sensitivity enhancement may reduce stability due to increased surface reactivity, whereas selectivity improvement via complex surface modifications can lead to slower response times. Similarly, miniaturization can enhance device portability but weaken signal intensity due to a reduced electroactive surface area, while increasing design complexity may compromise the system’s real-world reliability. These interdependent factors highlight the need for a holistic and strategic design, which is summarized in Supplementary Table S6.

### Critical research gaps and future directions

#### Unresolved technical challenges

The rapid development of nonenzymatic electrochemical sensors for neurotransmitter detection has revealed several unresolved research gaps that require focused investigation. One of them is the absence of standardized interference testing protocols for complex biological matrices (Mlambo et al. 2023). Current studies employ different experimental conditions, interferent concentrations, and sample compositions, preventing direct comparisons of sensor performance. This lack of standardization limits reproducibility and hinders the objective evaluation of selectivity, particularly for the application of sensors in real biological fluids such as blood, cerebrospinal fluid, or interstitial fluid. Establishing universally accepted testing protocols would significantly improve data comparability and accelerate the translation of laboratory-scale innovations into clinically relevant technologies.

Another major issue concerns the long-term stability of electrochemical sensors and the underlying mechanisms of material degradation. Although many sensor platforms demonstrate excellent short-term performance, prolonged exposure to electrochemical cycling, biofouling, and environmental stressors causes surface degradation, signal drift, and loss of sensitivity (Moulahoum and Ghorbanizamani 2024). The specific chemical, mechanical, and electrochemical pathways driving these degradation processes are not fully understood. Comprehensive studies focusing on stability mechanisms, degradation kinetics, and failure modes are therefore essential to design durable sensor materials for continuous or long-term monitoring.

Manufacturing scalability also represents a critical bottleneck in the advancement of nonenzymatic electrochemical sensors. While numerous designs perform well at the laboratory scale, consistent largescale production remains challenging. Variations in material synthesis, electrode modification techniques, and fabrication conditions can lead to significant inconsistencies across batches, ultimately affecting sensor reliability and cost-effectiveness (Sengupta 2025). Developing scalable manufacturing strategies that ensure uniform quality, reproducibility, and affordability is pivotal for the commercialization and widespread adoption of these sensing technologies.

In parallel, there is a growing need for predictive models to accurately forecast sensor performance in complex and dynamic environments. Most evaluations occur under controlled laboratory conditions that fail to capture the variability encountered in real biological systems. Predictive modeling approaches incorporating physicochemical interactions, matrix effects, and environmental fluctuations would provide valuable insights into sensor behavior and performance limitations. Such models could guide sensor optimization, reduce trial and error, and improve confidence in realworld applications.

Beyond these technical challenges, several emerging opportunities offer promising directions for future research. Integrating machine learning into electrochemical sensing platforms can significantly enhance selectivity and data interpretation. By analyzing complex signal patterns and compensating for interference, machine learning algorithms improve the discrimination of target neurotransmitters from multiple competing species. This data-driven approach is valuable in complex biological environments where traditional signal processing methods are insufficient. The development of biocompatible coatings is particularly important for implantable and *in vivo* sensing applications. Biocompatible surface modifications can reduce immune responses, minimize biofouling, and improve long-term sensor stability within biological tissues (Shaikh and Desai 2024). Advances in polymer science and surface engineering may allow creating coatings that not only protect sensor materials but also maintain high sensitivity and rapid response times over extended periods. Furthermore, wireless sensor networks open new possibilities for continuous and real-time neurotransmitter monitoring. Wireless integration facilitates remote data transmission, reduces the need for invasive sampling, and enables long-term monitoring in both clinical and research settings (Bakri et al. 2024). Such systems could clarify neurotransmitter dynamics in neurological disorders and personalized medicine.

Finally, personalized calibration algorithms tailored to individual patient variability represent a forward-looking research direction. Biological differences among individuals, such as variations in metabolism, tissue composition, and baseline neurotransmitter levels, can significantly influence sensor responses. Personalized calibration strategies, supported by adaptive algorithms, could enhance patient-specific measurement accuracy and reliability (Papi and Rastogi 2024). Overall, the field requires systematic comparative studies, standardized testing protocols, and concerted efforts toward clinical translation to fully realize the potential of nonenzymatic electrochemical sensors. Addressing unresolved technical challenges while exploring emerging opportunities is essential to bridge the gap between laboratory research and practical clinical implementation (Bakri et al. 2024; Melo et al. 2025; Moulahoum and Ghorbanizamani 2024).

## Neurotransmitter-specific detection: comparative analysis and technical challenges amino acid neurotransmitters

### Glutamate: key detection requirements and sensor performance

Glutamate is the predominant excitatory neurotransmitter in the mammalian central nervous system, serving as the primary signaling molecule for approximately 90% of brain neurons (Kaur et al. 2025; Samaripour 2025). Despite its major role in cognition, memory, learning, and synaptic plasticity, glutamate detection presents unique analytical challenges due to its low extracellular concentrations (2–40 μM) compared to synaptic cleft levels (up to 100 mM during neurotransmission) (Table 7) (Puranik 2024).

**Table 7:** Comparative performance of glutamate sensors

Aspect	Current status	Major barriers	Development priority	Timeline to clinical use
Analytical performance	Laboratory validated	Biological interference	High	2–3 years
Manufacturing scalability	Prototype level	Cost-effective production	Medium	3–5 years
Regulatory compliance	Pre-clinical	Safety/efficacy validation	High	5–7 years
Clinical integration	Concept stage	Healthcare workflow	Medium	7–10 years
Point-of-care deployment	Research phase	Miniaturization/stability	High	5–8 years

#### Critical performance analysis

Electrochemical sensors for glutamate detection must meet several fundamental requirements to ensure reliable and clinically relevant measurements. Sensitivity is an important parameter, as glutamate is typically present at physiological concentrations ranging from approximately 2 to 40 μM in extracellular fluids. Accurate detection within this range necessitates sensors with sub-micromolar limits of detection to reliably capture even subtle fluctuations in glutamate levels (Ali et al. 2023; Zeynaloo et al. 2021). Insufficient sensitivity may obscure early pathological changes or transient neurotransmitter dynamics, thereby limiting the diagnostic and analytical value of the sensor, particularly *in vivo*.

Selectivity is another major challenge in glutamate sensing due to the presence of structurally and electrochemically similar compounds in biological environments. Amino acids such as γ-aminobutyric acid, aspartate, and other endogenous species can generate interfering signals or compete for active sites on the electrode surface (Eskandarinezhad et al. 2022). Effective discrimination between glutamate and these coexisting molecules is essential to avoid false signals and data misinterpretation. Therefore, sensor designs must incorporate material engineering or surface modifications that isolate glutamate while suppressing responses from interfering analytes under complex physiological conditions.

Temporal resolution must also be carefully optimized. Real-time monitoring of neurotransmitter release and uptake requires rapid sensor response times to capture transient synaptic events. However, increasing response speeds often compromises signal stability, leading to higher noise or baseline drift. Consequently, an optimal balance between fast response kinetics and stable signal output is required for accurate and reproducible measurements over extended periods, especially for *in vivo* applications, where dynamic biological processes and environmental fluctuations can significantly affect sensor performance.

Recent advancements in nanostructured electrodes show substantial improvements in addressing these performance challenges. In particular, vertically aligned nickel nanowire array electrodes (NiNAE) and platinum-coated NiNAE (Pt/NiNAE) significantly boost electrocatalytic activity. These architectures dramatically increase the effective surface area, providing a higher density of active sites for glutamate oxidation. Additionally, the aligned nanowire structure facilitates more efficient electron transfer pathways between the analyte and the electrode, enhancing current responses and improving signal-to-noise ratios. As a result, these nanostructured electrodes enable real-time, *in vivo* glutamate detection with superior sensitivity, selectivity, and temporal resolution compared to conventional flat electrode surfaces (Ali et al. 2023; Zeynaloo et al. 2021).

From a clinical perspective, high-performance glutamate biosensors hold significant implications for neurological research and healthcare. Dysregulation of glutamate signaling is strongly associated with a range of neurological and neurodegenerative disorders, including schizophrenia, Parkinson’s disease, epilepsy, stroke, and amyotrophic lateral sclerosis. Abnormal glutamate levels can trigger excitotoxicity, synaptic dysfunction, and progressive neuronal damage. Therefore, real-time concentration monitoring offers valuable insights into disease mechanisms, progression, and treatment response. Advanced biosensors capable of continuous, accurate glutamate monitoring may also support the evaluation of therapeutic interventions, paving the way for more precise and personalized treatment strategies for neurological patients (Nicosia et al. 2024; Papi and Rastogi 2024).

### Biogenic amine neurotransmitters

#### Comparative analysis of detection challenges

Biogenic amines, characterized by amine groups without carboxyl functionalities, serve essential roles in neurotransmission across the central and peripheral nervous systems (Khushboo et al. 2022). The structural similarities among dopamine, serotonin, and norepinephrine necessitate sophisticated selectivity strategies (Supplementary Table S7).

#### Synthesis and metabolism pathway considerations

Biogenic amine neurotransmitters are synthesized through highly regulated biochemical pathways originating from specific amino acid precursors. A thorough understanding of these pathways is essential to designing rational electrochemical sensors. Dopamine is biosynthesized from tyrosine through sequential enzymatic steps involving tyrosine hydroxylase and aromatic L-amino acid decarboxylase, while serotonin is formed from tryptophan via tryptophan hydroxylase-mediated hydroxylation followed by decarboxylation. Norepinephrine is subsequently produced from dopamine by dopamine β-hydroxylase (Khushboo et al. 2022). These tightly interconnected metabolic pathways generate target neurotransmitters, intermediate compounds, and metabolic byproducts that coexist within the same biological environment. As a consequence, sensor systems must discriminate target analytes from structurally related intermediates and degradation products that can produce overlapping electrochemical signals.

Enzymatic regulation further complicates neurotransmitter detection and directly impacts sensor performance requirements. Enzymes such as monoamine oxidase and catechol-O-methyltransferase rapidly degrade catecholamines, significantly shortening their extracellular lifetime and reducing their concentration. In parallel, high-affinity reuptake transporters actively remove neurotransmitters from the synaptic cleft, limiting the temporal window during which extracellular concentrations are detectable. Additionally, vesicular storage and release dynamics introduce rapid, transient fluctuations in neurotransmitter levels, creating highly dynamic concentration profiles instead of steady-state conditions. These biological factors collectively demand electrochemical sensors with faster response times (typically < 1 s) and high temporal resolution to capture physiologically meaningful concentration changes. Without such characteristics, crucial neurochemical events may be missed or inaccurately recorded (Khushboo et al. 2022).

#### Clinical correlation and therapeutic implications

Alterations in biogenic amine neurotransmitter levels are strongly associated with a wide range of neurological and psychiatric disorders, making them clinically relevant targets for both diagnostic and therapeutic strategies. Imbalances in dopamine, serotonin, and norepinephrine signaling are linked to depression, schizophrenia, Parkinson’s disease, and anxiety disorders (Azizi 2022; Teleanu et al. 2022). These correlations have driven pharmacological interventions aimed at modulating neurotransmitter synthesis, release, reuptake, or degradation (Supplementary Table S8). In this context, direct, real-time monitoring of neurotransmitter levels is a powerful approach to evaluate disease progression and therapeutic efficacy. The integration of advanced sensing technologies with treatment strategies is promising for personalized medicine, enabling therapy optimization based on objective neurochemical data rather than relying solely on clinical symptoms or behavioral assessments (Azizi 2022).

### Dopamine: advanced detection strategies and clinical applications

#### Comprehensive performance analysis

Among biogenic amine neurotransmitters, dopamine has been most researched due to its central role in brain function and its favorable electrochemical behavior (Gopika and Saraswathyamma 2025). It plays a vital role in motor control, reward processing, and cognitive function, and its dysregulation is a hallmark of several neurological disorders. As summarized in Supplementary Table S9, dopamine exhibits well-defined redox activity that facilitates electrochemical detection. However, achieving selective and sensitive dopamine detection in complex biological matrices is limited by the presence of structurally similar compounds, high concentrations of electroactive interferents, and the heterogeneous chemical environment of brain tissue, all of which complicate accurate measurements. These factors necessitate advanced sensor designs that exhibit high performance under physiologically relevant conditions. chemical environment of brain tissue complicate accurate measurement. These factors necessitate advanced sensor designs capable of maintaining high performance under physiologically relevant conditions.

#### Mechanistic understanding and optimization strategies

The electrochemical detection of dopamine is fundamentally based on its irreversible oxidation at physiological pH, resulting in the formation of dopamine-o-quinone through a two-electron, two-proton transfer mechanism. This process typically occurs at an oxidation potential of approximately +0.2 V vs. Ag/AgCl, which provides a basis for selective detection. However, this potential is proximal to those of common interferents, such as ascorbic acid at around +0.1 V and uric acid at approximately +0.35 V (Gopika and Saraswathyamma 2025; Teleanu et al. 2022). Effective dopamine sensing, therefore, requires precise control of the applied potential to minimize interference while preserving sufficient signal intensity. Optimization strategies must carefully balance electrode material properties, surface modification approaches, and electrochemical operating conditions.

Electrode material affects conductivity, surface area, and long-term stability, all of which directly influence sensor sensitivity and durability (Carbone et al. 2025). Surface modification strategies are equally important, as they can enhance selectivity through molecular recognition or electrostatic effects without excessively compromising sensitivity. Optimization of the potential window suppresses interfering signals, while appropriate pH buffering can maintain physiological relevance and ensure accurate representation of *in vivo* conditions. Recent advances demonstrate that combining nanostructured materials with precisely engineered surface functionalization dramatically enhances performance, enabling near-single-molecule sensitivity while retaining selectivity in complex biological environments (Gupta et al. 2020; Gopika and Saraswathyamma 2025).

#### Clinical translation and real-world challenges

The translation of dopamine sensors from laboratory prototypes to clinically deployable devices involves numerous regulatory and practical challenges (Supplementary Table S10).These include compliance with medical device regulations, long-term safety and biocompatibility, operational robustness, and seamless clinical workflow integration (Sengupta 2025). Addressing these considerations is essential to move sensor technologies beyond proof-of-concept to achieve meaningful impact in patient care.

#### Advanced applications and future directions

Advanced dopamine-sensing technologies open new opportunities across clinical and research applications. In Parkinson’s disease, real-time dopamine monitoring helps to directly assess levodopa treatment effectiveness and track motor fluctuations for precise dose adjustments (Kawahata et al. 2024; Ramesh and Arachchige 2023). In addiction research, continuous dopamine measurements clarify reward pathway dysfunction and treatment responses. Similarly, objective dopamine monitoring aids in optimizing psychiatric therapies by providing quantitative feedback on antipsychotic medication effects (Azizi 2022). In neurosurgical settings, intraoperative dopamine monitoring can guide deep-brain stimulation procedures via real-time neurochemical feedback.

Future priorities include the miniaturization of implantable sensor systems while focusing on biocompatibility and wireless data transmission (Bakri et al. 2024). The development of multi-analyte platforms that can simultaneously detect dopamine, its metabolites, and related neurotransmitters will provide a more comprehensive view of neurochemical signaling (Qi et al. 2025). Additionally, integrating machine learning approaches will enhance pattern recognition, improve selectivity, and support the clinical interpretation of complex datasets. The establishment of standardized experimental and validation protocols is also critical to ensure reproducibility and enable cross-laboratory comparisons. The convergence of advances in nanotechnology, clinical validation studies, and regulatory framework development positions dopamine sensors for a substantial clinical impact within the next decade (Ali and Dholaniya 2022; Ramesh and Arachchige 2023; Kawahata et al. 2024).

#### Critical research gaps and standardization needs

Despite significant progress, systematic challenges persist across neurotransmitter sensor classes. The most urgent research need is the development of standardized interference-testing protocols to consistently evaluate sensor performance in biological matrices. Crossplatform validation studies are also required to enable meaningful comparisons between different sensing technologies and fabrication approaches. Furthermore, robust clinical correlation studies must establish clear relationships between sensor readings and patient outcomes to strengthen the clinical relevance of neurotransmitter monitoring. Long-term biocompatibility remains another critical concern, requiring implanted sensors to be evaluated for tissue response and functional stability over months or years (Shaikh and Desai 2024).

From a technology integration perspective, data standardization is essential to support multi-site clinical studies and large-scale data analysis. The implementation of rigorous quality control protocols will help ensure consistent sensor performance across manufacturing batches, while regulatory harmonization can streamline the approval processes for neurotransmitter monitoring devices. Systematic development in these areas will accelerate the translation of laboratory innovations into clinical applications, improving patient care through objective, real-time neurotransmitter monitoring (Mlambo et al. 2023).

## Nano-biosensors

**Figure 2:**
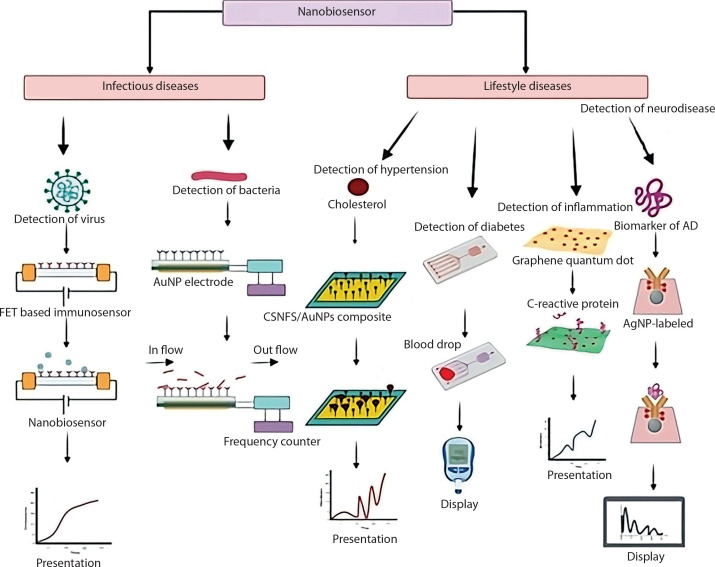
Various types of Nano-biosensors are applied in the biomedical field. Nano-biosensors are used to diagnose infectious diseases and lifestyle-related conditions. Field-effect transistors (FET)-based immunosensors are utilized to detect viral pathogens, while piezoelectric biosensors based on gold nanoparticles (AuNPs) detect bacterial pathogens. Nano-biosensors are also employed in the diagnosis of lifestyle-related diseases such as diabetes, hypertension, inflammatory diseases, and neurological disorders. Chitosan nanofiber scaffold (CSNFS)/AuNP composite tissue is used to detect blood cholesterol levels associated with hypertension, and electrochemical sensor chips are used to measure glucose levels from blood drops. Graphene Quantum Dots are used to identify C-reactive protein, a biomarker for inflammatory diseases. Various biomarkers are present in different neurological disorders and can be detected by electrochemical biosensors using silver nanoparticle (AgNP)-labeled antibodies. AD – Alzheimer’s disease

Nano-biosensors are highly specific analytical tools that detect diseases by recognizing biomarkers, supporting applications such as diagnosis, monitoring, treatment, and drug design (Ahmad et al. 2023; Das et al. 2024). These sensors assist in tissue engineering and aid targeted drug delivery in nanomedicine, where nanoscale size increases surface area and improves tissue response and implantation efficiency. In addition, nano-biosensors are applied to detect microbes in various samples, track metabolites in body fluids, and diagnose tissue-related diseases such as cancer. Beyond biomedical applications, nano-biosensors are also utilized in environmental analysis and food packaging, tracking, and security. Due to their ability to function effectively with minimal analyte concentrations, these sensors are valuable for early and accurate detection (Fritea et al. 2021). Figure 2 shows the working modes of various nano-biosensors and their applications in the biomedical field.

## Designing nano-biosensors

Nano-biosensors are advanced analytical devices that integrate nanotechnology with biomolecules to detect specific targets in biological samples (Barhoum et al. 2024). These sensors utilize nanoparticles, such as gold, carbon, or metal oxides, functionalized to bind with target bio-molecules (Philip and Kumar 2022; Sarakatsanou et al. 2023; Zhou et al. 2021). For example, AuNPs bind with biomolecules, producing detectable electrochemical or optical signals, while modification of their surfaces with antibodies or DNA aptamers further enhances detection specificity (Philip and Kumar 2022). The main advantages of nano-biosensors include high sensitivity, miniaturized size, and the ability to detect analytes at very low concentrations. Ongoing advances in nanomaterials and biotechnology continue to expand their applications in medicine, drug discovery, and environmental sciences (Padma et al. 2023).

In clinical and biomedical contexts, nano-biosensors can detect disease-related proteins, pathogens, nucleic acids, metabolites, and tumor antigens, thereby supporting early diagnosis and therapeutic development. Their high-affinity probes improve diagnostic speed and accuracy by converting biochemical or biological interactions into measurable electrical or optical signals (Fritea et al. 2021).

Nano-biosensors typically consist of three main components: a bioreceptor that recognizes the target analyte, a transducer that converts the recognition event into an electrochemical, optical, or other measurable signal, and an electronic processing unit that interprets the data (Ahmad et al. 2023). These devices can be categorized by technology type, including electrochemical, acoustic wave, nanowire, and nanotube-based biosensors (Ahmad et al. 2023; Das et al. 2024). Acoustic wave nanosensors generate mechanical waves from electrical signals using anisotropic materials; nanowire sensors detect changes in surface charge density; and nanotubes offer a platform for immobilizing biomolecules for better signal transduction (Hwang et al. 2020). Overall, nano-biosensors operate through electrochemical or optical mechanisms depending on their intended application (Hwang et al. 2020).

Nanomaterials play a crucial role in the development of nanoelectrodes for biosensors by enhancing surface area, conductivity, and overall biosensing performance. The commonly used nanomaterials include graphene, CNTs, CPs, and AuNPs, which improve electrochemical properties and sensitivity (Tran and Piro 2021). For instance, AuNP-modified electrodes offer a wider pH range and generate larger reaction currents, and are therefore effective for detecting biomarkers such as hydrogen peroxide, an indicator of oxidative stress (Tong et al. 2022). The selection of nanomaterials is application-specific, which forms the foundation of nano-biosensor design.

Nano-biosensors can be classified by the nanomaterials used, transduction mechanism, or the type of analyte being detected. They are fabricated using two primary strategies: top-down – breaks down bulk materials into nanoscale components, and bottom-up – assembles nanomaterials from atoms or molecules through physical and chemical processes. Both methods produce high-quality, cost-effective sensors, while precision engineering further improves the performance of sensors (Elugoke et al. 2020; Fritea et al. 2021; Ross and Bockstaele 2021; Urmi et al. 2024). Various types of nano biosensors are used in the biomedical field, as shown in Figure 2.

**Figure 3:**
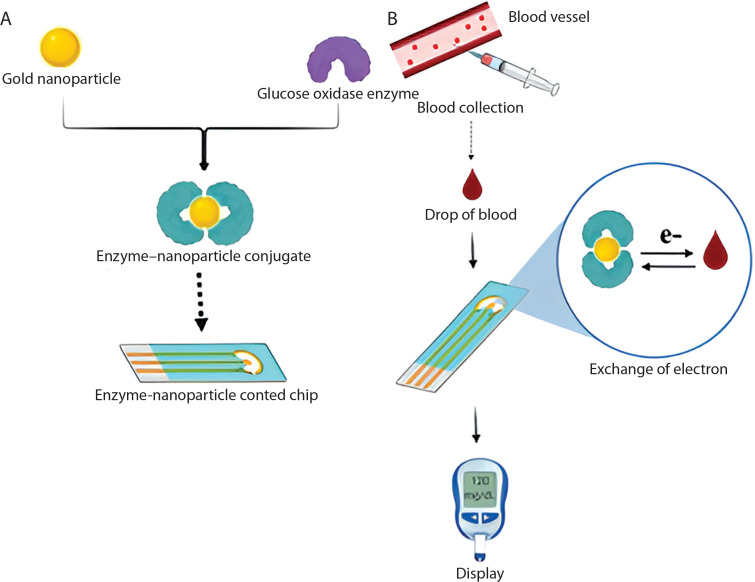
Electrochemical nano-biosensor mechanism. (A) Gold nanoparticles (NP) conjugated with glucose oxidase enzyme and an electric chip coated with the enzyme-NP conjugate. (B) Electron exchange occurs between the blood, collected from blood vessels, and the enzyme-NP conjugate coated on the electric chip through a chemical reaction

Advances in biosensor technology address analytical needs in disease diagnosis and treatment, therapy monitoring, and environmental protection (Barhoum et al. 2024; Sengupta 2025). Electrochemical nano-biosensors detect analytes through bioreceptor–analyte interactions on a nanomaterial-modified electrode, converting surface changes into measurable electrochemical signals (Li et al. 2019; Ramya et al. 2022; Tran and Piro 2021). These interactions cause measurable variations in electron transfer and charge accumulation, which directly correlate with analyte concentration. To optimize sensitivity and signal transduction, various nanomaterials, including nanoparticles, graphene, carbon dots, and CNTs, are incorporated into the working electrode (Gupta et al. 2020; Hui et al. 2022; Meskher et al. 2024; Sumitha and Xavier 2023). The electrochemical nano biosensor mechanism can be monitored on the display as shown in Figure 3.

## Conclusions

Nano-biosensors are powerful tools for detecting specific targets in biological samples. These sensors use nanoparticles, such as gold, carbon, or metal oxide nanoparticles, whose function can be modified to bind to specific targets. The main advantages of these sensors include high sensitivity, miniatured size, and the ability to quickly detect targets at ultralow concentrations. The development of biosensors primarily aims to meet analytical needs in disease treatment and applications such as environmental protection. For example, electrochemical nano-biosensors help determine patient conditions or monitor post-therapy responses. These sensors operate through interactions between a bioreceptor and an analyte, typically a biological entity associated with an electrode conjugated with nano-sized substances. With nanomaterials, such as nanoparticles, graphene, carbon dots, or CNTs, integrated in the electrode coating, these sensors can aid in highly sensitive and selective detection of neurotransmitters, which are crucial molecules in the nervous system. This review demonstrates that nano-biosensors offer significant potential for applications in neurological diagnosis and studies. Looking ahead, the adaptability of nano-biosensor technology promises to revolutionize fields such as immunology and nutrigenetics, paving the way for personalized medicine and advanced therapeutic monitoring.

## Supplementary Material


