# Supplementary material for: Trends in developing nanoparticle-enhanced electrochemical sensors for neurotransmitter detection: a review
Source: BioTechnologia (Pozn). 2026 Jun 27;107(2):181–204. doi: 10.5114/bta/221513 (PMC13409367; doi:10.5114/bta/221513)
Supplement: Supplementary file 1 [file BTA-107-2-221513-s1.pdf]

Supplementary Table S1. Selectivity Enhancement Approaches and Their Effectiveness

Strategy	Mechanism	Selectivity Improvement	Implementation Complexity	Cost Impact	Clinical Feasibility
Size exclusion membranes	Physical separation	3–5x	Low	Low	High
Surface functionalization	Chemical specificity	5–10x	Medium	Medium	Medium
Potential modulation	Electrochemical separation	2–4x	Low	Low	High
Molecularly imprinted polymers	Molecular recognition	10–20x	High	High	Low
Multi-electrode arrays	Differential detection	4–8x	Medium	Medium	Medium

Supplementary Table S2. Quantitative Interference Analysis

Interferent	Concentration in Blood (µM)	Signal Interference (%)	Mitigation Strategy	Effectiveness
Ascorbic Acid	50–100	15–40%	Nafion coating	80% reduction
Uric Acid	200–400	20–35%	pH optimization	70% reduction
Glucose	5000–8000	5–15%	Potential selection	90% reduction
Proteins	Variable	10–25%	BSA blocking	75% reduction

Supplementary Table S3. Clinical Translation Readiness Assessment

Aspect	Current Status	Major Barriers	Development Priority	Timeline to Clinical Use
Analytical Performance	Laboratory validated	Biological interference	High	2–3 years
Manufacturing Scalability	Prototype level	Cost-effective production	Medium	3–5 years
Regulatory Compliance	Pre-clinical	Safety/efficacy validation	High	5–7 years
Clinical Integration	Concept stage	Healthcare workflow	Medium	7–10 years
Point-of-care deployment	Research phase	Miniaturization/stability	High	5–8 years

Supplementary Table S4. Real-World Performance Limitations of Non-Enzymatic Sensors

Limitation Category	Specific Issues	Impact on Practical Performance
**Stability Challenges**	- Signal drift over time (5–25% per week) - Electrode fouling in biological matrices - Strict storage requirements (−20°C to +4°C)	Reduces long-term accuracy; increases maintenance needs; limits field and clinical usability.
**Reproducibility Issues**	- Batch-to-batch variations (15–30% RSD) - Inter-electrode differences (10–25% RSD) - Lack of fabrication standardization	Leads to inconsistent results across sensors; hampers large-scale production and clinical validation.
**Environmental Sensitivity**	- Temperature effects (2–5% per °C) - Strong pH dependency in biological fluids - Ionic strength interference	Causes fluctuating sensor responses; complicates measurements in variable or dynamic environments.

Supplementary Table S5. Data Management and Regulatory Considerations

Category	Items
**Data Management**	• Real-time data transmission capabilities• Cloud-based analytics for pattern recognition• Integration with electronic health records• AI-assisted interpretation algorithms
**Regulatory Considerations**	• FDA approval pathways for digital therapeutics• Data privacy and security compliance• Clinical validation requirements• Quality management systems

Supplementary Table S6. Rational Sensor Design Framework

Category	Key Considerations	Description
**Material Selection Criteria**	Sensitivity Requirements	Ensure limits of detection (LOD) align with physiological neurotransmitter concentrations.
**Design Trade-offs**	Selectivity Needs	Evaluate potential interference from endogenous biological compounds.
	Stability Demands	Match material durability with the intended application duration and storage conditions.
	Cost Constraints	Balance sensor performance with affordability for practical and clinical use.
	Sensitivity vs. Stability	Higher sensitivity often decreases long-term stability due to increased surface reactivity.
	Selectivity vs. Response Time	Improved selectivity through surface engineering can slow sensor response.
	Miniaturization vs. Signal Strength	Smaller electrode dimensions lower current output and reduce signal intensity.
	Complexity vs. Reliability	Additional design features may compromise robustness in practical environments.

Supplementary Table S7. Biogenic Amine Detection Performance Comparison

Neurotransmitter	Physiological Range (nM–µM)	Electrochemical Potential (V vs. Ag/AgCl)	Major Interferences	Best Achieved LOD (nM)	Clinical Significance
Dopamine	10 nM – 1 µM	+0.15 to +0.25	NE, DOPAC, AA, UA	5	Parkinson's, addiction
Serotonin	50 nM – 2 µM	+0.35 to +0.45	Tryptophan, 5-HIAA	25	Depression, anxiety
Norepinephrine	5 nM – 800 nM	+0.10 to +0.20	DA, epinephrine	10	ADHD, hypertension
Epinephrine	1 nM – 500 nM	+0.05 to +0.15	NE, DA	8	Stress response

Supplementary Table S8. Clinical Correlations and Therapeutic Targeting

Disorder	Primary NT Imbalance	Diagnostic Markers	Current Therapies	Sensor Application Potential
Depression	↓ Serotonin, ↓ NE, ↓ DA	Subjective assessment	SSRIs, SNRIs, MAOIs	Therapy monitoring, dose optimization
Parkinson's Disease	↓↓ Dopamine	Motor symptoms, DaTscan	Levodopa, DA agonists	Disease progression tracking
Schizophrenia	↑ Dopamine (mesolimbic)	Positive/negative symptoms	Antipsychotics	Treatment response prediction
ADHD	↓ Norepinephrine, ↓ DA	Behavioral assessment	Stimulants, atomoxetine	Medication titration

Supplementary Table S9. State-of-the-Art Dopamine Sensor Performance

Sensor Configuration	LOD (nM)	Linear Range (µM)	Selectivity Ratio (DA: AA)	Real Sample Performance	Stability (days)	Reference
AuNPs/graphene/GCE	3.2	0.01–100	1500:01:00	Serum: 92% recovery	30	(Gopika & Saraswathyamm 2025)
MoS_2/rGO composite	5.8	0.02–80	800:01:00	Urine: 95% recovery	25	(Nimgampalle et al. 2023)
Polymer/CNT hybrid	2.1	0.005–50	2000:01:00	CSF: 88% recovery	20	(Rosikon et al. 2023)
Pt nanoparticles/PEDOT	4.5	0.01–120	1200:01:00	Blood: 90% recovery	35	(Samaripour 2025)

Supplementary Table S10. Clinical Translation Readiness Assessment for Dopamine Sensors

Aspect	Current Status	Regulatory Requirements	Technical Barriers	Timeline
Analytical validation	Laboratory proven	ISO 15197 compliance	Matrix effects, calibration	1-2 years
Clinical validation	Pilot studies	FDA 510(k) or PMA	Patient variability, correlation with symptoms	3-5 years
Manufacturing	Prototype scale	GMP compliance, quality systems	Reproducibility, cost control	2-4 years
